# Discordant risk factors between pancreatic neuroendocrine neoplasms and pancreatic ductal adenocarcinoma

**DOI:** 10.1530/ERC-24-0142

**Published:** 2025-03-01

**Authors:** Shruti Chandra, Thorvardur R Halfdanarson, Erin E Carlson, Kari G Rabe, Amit Mahipal, Shounak Majumder, William R Bamlet, Masayasu Horibe, Sri Harsha Tella, Omair Shariq, Ryan M Carr, Sean P Cleary, Ann L Oberg, Samuel O Antwi

**Affiliations:** ^1^ Department of Quantitative Health Sciences, Mayo Clinic, Rochester, Minnesota, USA; ^2^ Division of Medical Oncology, Department of Internal Medicine, Mayo Clinic, Rochester, Minnesota, USA; ^3^ Department of Oncology, Seidman Cancer Center, Case Western Reserve University, Cleveland, Ohio, USA; ^4^ Division of Gastroenterology and Hepatology, Department of Internal Medicine, Mayo Clinic, Rochester, Minnesota, USA; ^5^ Division of Gastroenterology and Hepatology, Department of Internal Medicine, Keio University School of Medicine, Tokyo, Japan; ^6^ Department of Hematology and Oncology, Mayo Clinic, Rochester, Minnesota, USA; ^7^ Department of General Surgery, Mayo Clinic, Rochester, Minnesota, USA; ^8^ Division of Hepatobiliary and Pancreatic Surgery, Mayo Clinic, Rochester, Minnesota, USA; ^9^ Division of Epidemiology, Department of Quantitative Health Sciences, Mayo Clinic, Jacksonville, Florida, USA; ^10^ Division of Gastroenterology and Hepatology, Department of Internal Medicine, Mayo Clinic, Jacksonville, Florida, USA

**Keywords:** pancreatic neuroendocrine neoplasms, neuroendocrine cancer, risk factors, panNEN, panNET, nets, NEC

## Abstract

Pancreatic neuroendocrine neoplasm (panNEN) is a rare malignancy and the second most common type of pancreatic cancer after pancreatic ductal adenocarcinoma (PDAC), but its etiology is poorly understood. We investigated whether the risk factors of panNEN are concordant with those known for PDAC. We performed the largest case-control study to date on panNENs, comprising 927 sporadic nonfunctional panNEN cases and 1807 frequency-matched controls, using data from the Mayo Clinic Biospecimen Resource for Pancreas Research. We assessed associations for obesity, first-degree family history of pancreatic cancer, cigarette smoking, overall type II diabetes mellitus (T2DM), new-onset T2DM (<1 year before panNEN diagnosis), longstanding T2DM (≥5 years), alcohol intake and aspirin use. Multivariable logistic regression was used to calculate odds ratios and 95% confidence intervals (CIs). Our results show that overall T2DM (OR = 1.71, 95% CI: 1.37–2.14) and new-onset T2DM (OR = 2.65, 95% CI: 1.92–3.69) are associated with higher odds of panNEN, but not longstanding T2DM (OR = 1.29, 95% CI: 0.94–1.75). A non-significant elevated odds of panNEN was observed among participants with a positive family history of pancreatic cancer (OR = 1.44, 95% CI: 0.96–2.14). Alcohol use was inversely related to panNEN (OR = 0.52, 95% CI: 0.42–0.66, ever-*vs*-never). No association was observed for smoking, obesity or aspirin use. These findings indicate that overall T2DM and new-onset T2DM are associated with higher odds of panNEN. Unlike PDAC, alcohol use was inversely related to panNEN, and we found no associations for cigarette smoking, obesity or aspirin use. These results indicate differences in the risk factor profiles of panNEN and PDAC.

## Introduction

Pancreatic neuroendocrine neoplasms (panNENs) are the second most common type of pancreatic cancer, comprising about 2% of cases, with pancreatic ductal adenocarcinoma (PDAC) being the most common type and constitutes roughly 95% of cases (Batcher *et al.* 2011, Halfdanarson *et al.* 2014, Antwi *et al.* 2017). Two major distinctions between panNENs and PDACs are that while panNENs originate from the islet cells in the endocrine pancreas, PDACs originate from the acinar cells in the exocrine pancreas, and panNENs have an overall better prognosis than PDACs (Anderson & Bennett 2016, Antwi *et al.* 2017, Yadav *et al.* 2018). Although panNENs are rare malignancies, their incidence has been rising in the United States and many parts of the world over the past few decades (Halfdanarson *et al.* 2008, Yao *et al.* 2008, Milan & Yeo 2012, Xu *et al.* 2021, Sonbol *et al.* 2022). In the US, the incidence of panNENs rose from 1.1 per million population in 1973 to 2.7 per million in 2000 to 10 per million in 2016 (Halfdanarson *et al.* 2008, Sonbol *et al.* 2022). Reasons for the rising incidence of panNENs are not entirely clear but have been attributed to various factors, including improvements in diagnostic technologies, increased use of cross-sectional imaging modalities resulting in an increase in incidental diagnoses, increased awareness and a rise in the prevalence of potential risk factors (Yao *et al.* 2008, Chan *et al.* 2018). Unlike PDAC, where the genetic and non-genetic risk factors are well established (Antwi *et al.* 2016, Antwi *et al.* 2017, Hu *et al.* 2018, Klein *et al.* 2018), the risk factors of panNENs are poorly understood.

A thorough literature search shows that to date, only seven studies have investigated associations between exposure to known or suspected cancer-predisposing factors and risk of panNEN development (Hassan *et al.* 2008, Capurso *et al.* 2009, Halfdanarson *et al.* 2014, Ben *et al.* 2016, Valente *et al.* 2017, Giraldi *et al.* 2021, Feola *et al.* 2022). Except for type II diabetes mellitus (T2DM) and family history of cancer, there are substantial inconsistencies across studies on whether obesity, cigarette smoking and alcohol use are associated with a higher risk of panNEN (Hassan *et al.* 2008, Capurso *et al.* 2009, Halfdanarson *et al.* 2014, Ben *et al.* 2016, Valente *et al.* 2017, Giraldi *et al.* 2021, Feola *et al.* 2022). Only two (Halfdanarson *et al.* 2014, Feola *et al.* 2022) out of the seven studies found an association between obesity and panNEN, and only one study (Ben *et al.* 2016) found an association between cigarette smoking and panNEN. Results from studies investigating the association between alcohol intake and panNEN risk have varied widely, ranging from a 4.8-fold higher risk of panNEN (Capurso *et al.* 2009) to a 44% lower risk of panNEN (Halfdanarson *et al.* 2014) to null findings in multiple studies (Hassan *et al.* 2008, Ben *et al.* 2016, Valente *et al.* 2017, Giraldi *et al.* 2021, Feola *et al.* 2022). Importantly, these studies were universally limited by small numbers of panNEN cases (*n* = 75–360), which may explain the conflicting results.

Substantially more epidemiological studies have investigated the risk factors of PDAC (reviewed in Antwi *et al.* (2017), Klein (2021), Lowenfels & Maisonneuve (2006)). Overall, these studies show consistent evidence that obesity, T2DM, a positive first-degree family history of pancreatic cancer, heavy alcohol intake and cigarette smoking are associated with higher PDAC risk (Lowenfels & Maisonneuve 2006, Genkinger *et al.* 2009, Antwi *et al.* 2016, Midha *et al.* 2016, Antwi *et al.* 2017, Carreras-Torres *et al.* 2017, Klein 2021, Antwi *et al.* 2022). Studies also suggest that regular aspirin use may reduce risk for PDAC development (Tan *et al.* 2011, Streicher *et al.* 2014, Risch *et al.* 2017, Sun *et al.* 2019).

Given the rising incidence of panNEN and its poorly understood etiology, there is a pressing need to clarify the risk factors of this rare malignancy to improve strategies for risk prevention, risk stratification and to enhance early detection efforts. The prior studies on panNEN risk factors suggest a potentially shared etiology between panNEN and PDAC. Hence, we performed the largest study to date on panNEN risk assessment to verify whether the following risk factors of PDAC are also associated with risk for panNEN: longstanding T2DM, new-onset T2DM, obesity, cigarette smoking, first-degree family history of pancreatic cancer, heavy alcohol intake and regular aspirin use.

## Materials and methods

### Data source and study population

The study was reviewed and approved by the Mayo Clinic Institutional Review Board, and all participants provided written informed consent. Data were obtained from the Mayo Clinic Biospecimen Resource for Pancreas Research, a prospective patient registry that was supported by the Mayo Clinic Specialized Program of Research Excellence (SPORE) in pancreatic cancer. Details of the design and methods used for patient recruitment have been described previously (Antwi *et al.* 2015, 2016, 2019, 2022). In brief, the registry utilizes an ultra-rapid case ascertainment process to identify pancreatic cancer patients and has continuously enrolled patients since October 2000. Patients are approached for consent to participate in the registry studies by telephone ahead of their clinic visits for evaluation of suspected pancreatic cancer or during the initial clinic appointment for suspicion of pancreatic cancer. In all, 1,886 patients with panNEN diagnosed at Mayo Clinic between October 2000 and August 2023 were approached for recruitment, among whom 1,298 consented to the study and were enrolled in the registry, a response rate of 69%. Among the enrolled patients, 55% were enrolled within 30 days of panNEN diagnosis, with an average of 16 days between first contact and enrollment.

### Case ascertainment

**Figure 1:**
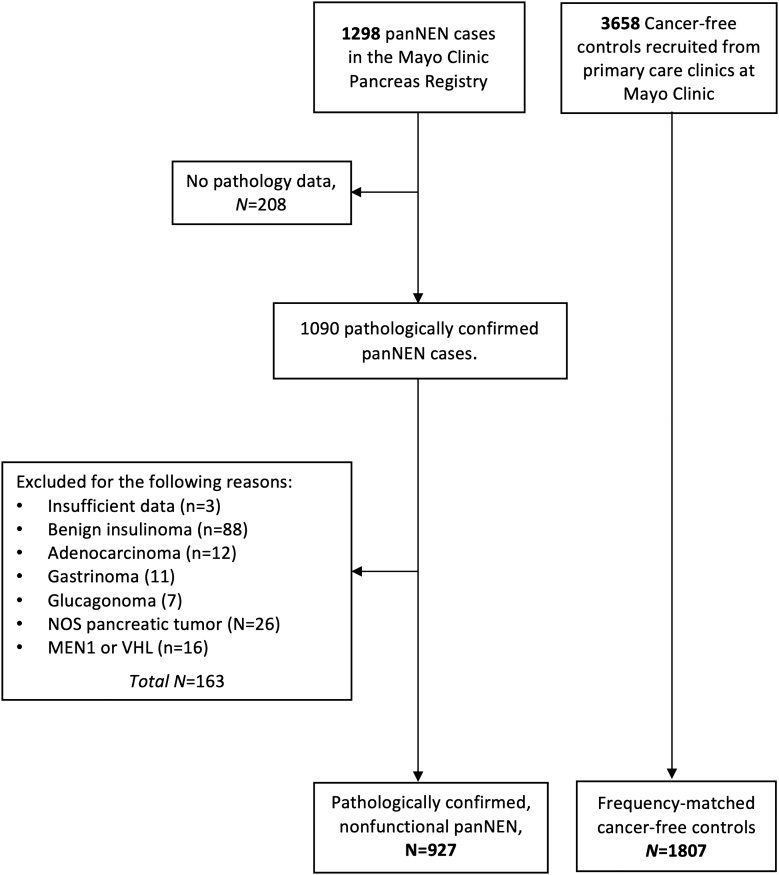
Flowchart of sample selection for cases and controls. The flowchart shows reasons for the exclusion of panNEN cases from the initial sample of 1,298 patients. We frequency-matched 927 panNEN cases to 1,807 cancer-free controls based on age (5-year intervals), sex and region of residence (Midwest, other) for final analyses. Abbreviations: panNEN, pancreatic neuroendocrine neoplasms; NOS, not otherwise specified.

Pathology reports were reviewed for all cases by a subspecialist physician for diagnosis coding, and clinical information specific to the diagnosis of panNEN was abstracted from clinical records by trained abstractors. To reduce disease heterogeneity, we restricted cases to pathologically confirmed sporadic nonfunctional panNEN. Specifically, of the 1,298 consented patients, we selected 1,090 cases with pathologically confirmed diagnoses of panNEN, among which we excluded three cases with insufficient data. We also excluded 88 cases with benign insulinoma, 12 cases with adenocarcinoma, 11 with gastrinoma, seven with glucagonoma, 26 non-specific panNEN (otherwise unspecified) and 16 cases with clinical diagnoses of *MEN1* or von Hippel-Lindau syndrome. After these exclusions, 927 cases of panNEN remained for analyses (Fig. 1 and Table 1).

**Table 1:** Descriptive statistics of study participants and univariable analyses of potential risk factors for nonfunctional sporadic panNENs.

Risk factors	Cases (*n* = 927)	Controls (*n* = 1807)		Unadjusted OR (95% CI)^b^
	*n* (%)	*n* (%)	*P*-value^a^	
Age, years (mean ± SD)	59.2 ± 12.7	60.1 ± 11.7	0.22	0.99 (0.99–1.00)
Sex			0.88	
Female	381 (41)	748 (41)		(Ref)
Male	546 (59)	1,059 (59)		1.01 (0.86–1.19)
Region of residence			0.48	
Midwest	519 (56)	1,037 (57)		(Ref)
Other	408 (44)	770 (43)		1.06 (0.90–1.24)
Race			0.31	
White	896 (97)	1759 (97)		(Ref)
Other	31 (3)	48 (3)		1.27 (0.79–2.00)
BMI, kg/m^2^			0.01	
≤24.9	238 (26)	533 (30)		(Ref)
25–29.9	368 (40)	741 (41)		1.11 (0.91–1.35)
≥30	321 (35)	533 (30)		1.35 (1.10–1.66)
5-unit increase (mean ± SD)	28.8 ± 5.8	28.2 ± 5.8	<0.001	1.09 (1.02–1.16)
Continuous (mean ± SD)	28.8 ± 5.8	28.2 ± 5.8	<0.001	1.02 (1.00–1.03)
Family history of pancreatic cancer^c^			0.18	
No	882 (95)	1739 (96)		(Ref)
Yes	45 (5)	68 (4)		1.30 (0.88–1.91)
Smoking history			0.16	
Never	562 (61)	1,113 (62)		(Ref)
Former	305 (33)	609 (34)		0.99 (0.84–1.18)
Current	60 (7)	85 (5)		1.40 (0.99–1.97)
Pack-years of smoking			0.94	
Never smokers	629 (68)	1,209 (67)		(Ref)
<10	119 (13)	231 (13)		0.99 (0.78–1.26)
10–19	59 (6)	121 (7)		0.94 (0.67–1.29)
≥20	120 (13)	246 (14)		0.94 (0.74–1.19)
Pack-years of smoking within smoking category			0.23	
Never	629 (68)	1,209 (67)		(Ref)
Former				
<10	110 (12)	227 (13)		0.93 (0.73–1.19)
10–19	53 (6)	111 (6)		0.92 (0.65–1.28)
≥20	97 (11)	207(12)		0.90 (0.69–1.16)
Current				
<10	9 (1)	4 (0.2)		4.32 (1.40–16.0)
10–19	6 (0.6)	10 (0.6)		1.15 (0.39–3.12)
≥20	23 (3)	39 (2)		1.13 (0.66–1.90)
History of T2DM			<0.001	
No	711 (77)	1,542 (85)		(Ref)
Yes	216 (23)	265 (15)		1.77 (1.45–2.16)
Duration of T2DM^d^			<0.001	
No T2DM	711(77)	1,542 (85)		(Ref)
≤1 year	108 (12)	79 (4)		2.96 (2.19–4.03)
1–4 years	26 (3)	46 (3)		1.23 (0.74–1.98)
≥5 years	82 (9)	140 (8)		1.27 (0.95–1.69)
Number of aspirin pills taken regularly			0.02	
Non-users/<1 per month	661 (71)	1,196 (66)		(Ref)
1–2 per day	219 (24)	502 (28)		0.79 (0.66–0.95)
≥3 per day	47 (5)	109 (6)		0.78 (0.54–1.11)
Ever alcohol use			<0.001	
No	173 (19)	247 (14)		(Ref)
Yes	501 (54)	1,419 (79)		0.50 (0.41–0.63)
Unknown^e^	253 (27)	141 (8)		—
Alcoholic drinks per day			<0.001	
Non-users	173 (19)	247 (14)		(Ref)
<1	349 (38)	984 (55)		0.50 (0.40–0.64)
1–2	103(11)	236 (13)		0.62 (0.46–0.84)
≥3	49 (5)	199 (11)		0.35 (0.24–0.50)
Unknown^e^	253 (27)	141 (8)		—
Ki-67 values				
≤2	156 (34.8%)	—	—	—
3–20	230 (51.3%)	—	—	—
>20	62 (13.8%)	—	—	—
Missing	479	—	—	—
Tumor stage				
I NOS	112 (12.6%)	—	—	—
IA	115 (13.0%)	—	—	—
IB	115 (13.0%)	—	—	—
II NOS	51 (5.7%)	—	—	—
IIA	44 (4.9%)	—	—	—
IIB	81 (9.1%)	—	—	—
III	24 (2.7%)	—	—	—
IV	346 (39.0%)	—	—	—
Missing	39	—	—	—

^a^ *P*-values comparing risk factor distribution between cases and controls using Wilcoxon’s test for continuous variables and chi-square test for categorical variables.

^b^ Each risk factor was modeled separately.

^c^ Family history of either PDAC or pancreatic neuroendocrine neoplasms.

^d^ Duration of T2DM before the diagnosis of pancreatic neuroendocrine neoplasms for cases or before recruitment for controls.

^e^ A separate category was created for those with unknown alcohol use history and was included in the logistic regression model. We did not provide results for this category as it cannot be interpreted or generalized to a particular population group.

Abbreviations: PDAC, pancreatic ductal adenocarcinoma; BMI, body mass index; CI, confidence interval; T2DM, type II diabetes mellitus; NOS, not otherwise specified; OR, odds ratio; panNEN, pancreatic neuroendocrine neoplasms; SD, standard deviation.

### Controls selection

We have previously provided details on the recruitment of controls (Antwi *et al.* 2015, 2016). In brief, controls were recruited from primary care clinics at the Mayo Clinic during regular wellness check visits. The controls did not have a personal history of any cancer type (except nonmelanoma skin cancer) or a personal history of pancreatitis. In all, 3,658 cancer-free controls were recruited from the primary care clinics. We frequency-matched up to two controls to each case based on age (5-year intervals), sex and region of residence (Midwest, other), resulting in 1,807 cancer-free controls being matched to the 927 panNEN cases for analyses (Fig. 1).

### Data collection and variable classification

The cases and controls completed identical risk factor questionnaires that included detailed questions on participant demographics, personal and family health history, lifestyle factors, including smoking history, alcohol and aspirin use. Data on age at diagnosis for cases and age at recruitment for controls and sex, were obtained from the risk factor questionnaires and verified with medical records. Information that was missing from the risk factor responses was abstracted from participants’ medical records. We used self-reported usual adult weight and height to categorize body mass index (BMI) as normal (≤24.9 kg/m^2^), overweight (25–29.9 kg/m^2^) or obese (≥30 kg/m^2^). Individuals who reported smoking <100 cigarettes in their lifetime were considered non-smokers and smoking history was categorized as never, former, current and as pack-years of smoking (<10 pack-years, 10–19 pack-years and ≥20 pack-years). We further categorized smoking history as pack-years of smoking within smoking categories (never, former with <10 pack-years, former with 10–19 pack-years, former with ≥20 pack-years, current with <10 pack-years, current with 10–19 pack-years and current with ≥20 pack-years). The participants also reported a first-degree family history of pancreatic cancer (no distinction between PDAC or panNEN; yes or no) and a personal history of T2DM diagnosis and duration of T2DM. We classified the presence of T2DM as yes or no and the duration of T2DM as follows: ≤1 year (new-onset T2DM), 1–4 years and ≥5 years (longstanding T2DM) (Antwi *et al.* 2016). Alcohol use was classified as yes or no and according to the number of alcoholic drinks consumed per day: non-users, <1 drink per day, 1–2 drinks per day and ≥3 drinks per day. In addition, we assessed the number of aspirin pills taken per day: non-users or <1 per month, 1–2 per day and ≥3 per day.

### Statistical analyses

Demographic, lifestyle and clinical factors were compared between the cases and controls using Wilcoxon’s test for continuous variables and chi-square tests for categorical variables. Odds ratios (ORs) and 95% confidence intervals (CIs) were calculated using unconditional logistic regression. We assessed associations between the following known PDAC risk factors and the odds of panNEN: BMI, first-degree family history of pancreatic cancer, smoking history, pack-years of smoking, pack-years of smoking within smoking category, T2DM, duration of T2DM, alcohol use, number of alcoholic drinks consumed per day, and number of aspirin pills taken regularly, as shown in Tables 1 and 2. Analyses were performed using univariable, minimally adjusted and fully adjusted models. For the minimally adjusted models, we adjusted for the matching factors (age, sex and region of residence). The fully adjusted models included additional adjustments for race, BMI, first-degree family history of pancreatic cancer, smoking status, T2DM and alcohol use. Each of these risk factors was adjusted for in models that did not examine the effect of that factor. As an example, because we have more than one BMI variable, we assessed the effect of each BMI variable separately while adjusting for the other risk factors. In sensitivity analyses, we repeated these analyses among participants with non-missing information in fully adjusted models (cases: *n* = 674, controls: *n* = 1,666; Supplementary Table S1 (see section on Supplementary materials given at the end of the article)). All statistical tests were two-sided, and *P*-values <0.05 or CIs that do not include ‘1’ were considered statistically significant. Analyses were performed using SAS version 9.4 (SAS Inc, USA).

**Table 2:** Minimally- and multivariable-adjusted analyses for potential risk factors of nonfunctional sporadic panNENs.

Risk factors	Cases (*n* = 927)	Controls (*n* = 1807)	Minimally-adjusted OR (95% CI)^a^	Fully-adjusted OR (95% CI)^b^
Race				
White	896 (97)	1759 (97)	(Ref)	(Ref)
Other	31 (3)	48 (3)	1.22 (0.76–1.93)	0.93 (0.56–1.52)
BMI, kg/m^2^				
≤24.9	238 (26)	533 (30)	(Ref)	(Ref)
25–29.9	368 (40)	741 (41)	1.14 (0.93–1.40)	1.07 (0.86–1.32)
≥30	321 (35)	533 (30)	1.37 (1.11–1.70)	1.05 (0.83–1.32)
5-unit increase	28.8 ± 5.8	28.2 ± 5.8	1.09 (1.02–1.16)	0.99 (0.92–1.07)
Continuous	28.8 ± 5.8	28.2 ± 5.8	1.02 (1.00–1.03)	1.00 (0.98–1.01)
Family history of pancreatic cancer^c^				
No	882 (95)	1739 (96)	(Ref)	
Yes	45 (5)	68 (4)	1.33 (0.90–1.94)	1.44 (0.96–2.14)
Smoking history				
Never smokers	562 (61)	1,113 (62)	(Ref)	
Former	305 (33)	609 (34)	1.02 (0.85–1.21)	1.18 (0.98–1.42)
Current	60 (7)	85 (5)	1.37 (0.96–1.93)	1.30 (0.89–1.87)
Pack-years of smoking				
Non-smokers	629 (68)	1,209 (67)	(Ref)	(Ref)
<10	119 (13)	231 (13)	1.01 (0.79–1.28)	1.28 (0.99–1.65)
10–19	59 (6)	121 (7)	0.96 (0.69–1.33)	1.19 (0.84–1.66)
≥20	120 (13)	246 (14)	0.96 (0.75–1.23)	1.15 (0.89–1.48)
Pack-years of smoking within smoking category				
Never	629 (68)	1,209 (67)	(Ref)	(Ref)
Former				
<10	110 (12)	227 (13)	0.95 (0.74–1.22)	1.23 (0.94–1.59)
10–19	53 (6)	111 (6)	0.94 (0.66–1.32)	1.19 (0.83–1.69)
≥20	97 (11)	207(12)	0.93 (0.71–1.21)	1.10 (0.83–1.46)
Current				
<10	9 (1)	4 (0.2)	4.19 (1.35–15.56)	3.31 (1.00–12.92)
10–19	6 (0.6)	10 (0.6)	1.12 (0.38–3.03)	1.14 (0.36–3.26)
≥20	23 (3)	39 (2)	1.12 (0.65–1.88)	1.36 (0.78–2.33)
History of T2DM				
No	711 (77)	1,542 (85)	(Ref)	(Ref)
Yes	216 (23)	265 (15)	1.83 (1.49–2.24)	1.71 (1.37–2.14)
Duration of T2DM^d^				
No T2DM	711(77)	1,542 (85)	(Ref)	(Ref)
<1 year	108 (12)	79 (4)	3.00 (2.21–4.08)	2.65 (1.92–3.69)
1–4 years	26 (3)	46 (3)	1.26 (0.76–2.04)	1.28 (0.76–2.12)
≥5 years	82 (9)	140 (8)	1.33 (0.99–1.77)	1.29 (0.94–1.75)
Number of aspirin pills taken regularly				
Non-users/<1 per month	661 (71)	1,196 (66)	(Ref)	(Ref)
1–2 per day	219 (24)	502 (28)	0.80 (0.66–0.97)	1.04 (0.85–1.27)
≥3 per day	47 (5)	109 (6)	0.78 (0.54–1.10)	1.06 (0.73–1.52)
Ever alcohol use				
No	173 (19)	247 (14)	(Ref)	(Ref)
Yes	501 (54)	1,419 (79)	0.51 (0.41–0.63)	0.52 (0.42–0.66)
Unknown^e^	253 (27)	141 (8)	—	—
Alcoholic drinks per day				
Non-users	173 (19)	247 (14)	(Ref)	(Ref)
<1	349 (38)	984 (55)	0.51 (0.41–0.65)	0.52 (0.42–0.69)
1–2	103(11)	236 (13)	0.62 (0.46–0.84)	0.65 (0.47–0.88)
≥3	49 (5)	199 (11)	0.35 (0.24–0.50)	0.36 (0.25–0.53)
Unknown^e^	253 (27)	141 (8)	—	—

^a^ Adjusting for matching factors: age (continuous), sex and region of residence (Midwest or other).

^b^ Additional adjustment for race (White or other), BMI (continuous), family history of pancreas cancer (yes or no), smoking status (never, former or current), T2DM (yes or no) and alcohol use (yes or no). We did not adjust for any of the BMI variables in models examining associations for BMI. Similarly, we did not adjust for any of the diabetes variables in models examining associations for diabetes; same with alcohol, smoking and aspirin use.

^c^ Family history of PDAC or pancreatic neuroendocrine neoplasms.

^d^ Duration of T2DM before the diagnosis of pancreatic neuroendocrine neoplasms for cases or before recruitment for controls.

^e^ A separate category was created for those with unknown alcohol use history and was included in the logistic regression models. We did not provide results for this category as it cannot be interpreted or generalized to a particular population group.

Abbreviations: PDAC, pancreatic ductal adenocarcinoma; BMI, body mass index; CI, confidence interval; T2DM, type II diabetes mellitus; OR, odds ratio; panNEN, pancreatic neuroendocrine neoplasms.

## Results

Descriptive statistics of the 927 nonfunctional sporadic panNEN cases and the 1807 cancer-free controls are shown in Table 1. Because of the frequency matching, the cases and controls did not differ by age, sex or region of residence. They also did not differ by race, first-degree family history of pancreatic cancer or smoking history. However, the cases had a higher percentage of individuals with obesity (BMI ≥30 kg/m^2^) than controls. Cases were also more likely than controls to have a diagnosis of T2DM, new-onset T2DM (≤12 months before panNEN diagnosis) and slightly more likely to have longstanding T2DM (≥5 years before panNEN diagnosis). Regular aspirin use and alcohol use were more frequently reported by controls than cases. Tumor characteristics of the cases have also been provided.

Table 1 further shows results of the univariable analyses. No association was found between race and panNEN, but it is important to note that the study participants were predominately White (97%). Compared to individuals with normal BMI, those with obesity had 35% higher odds of panNEN (OR = 1.35, 95% CI: 1.10–1.66; OR_5-unit increase_ = 1.09, 95% CI: 1.02–1.16; OR_continuous_ = 1.02, 95% CI: 1.00–1.03). Furthermore, a non-significant elevated odds of panNEN was observed among participants with a positive first-degree family history of pancreatic cancer (OR = 1.30, 95% CI: 0.88–1.91). The association between smoking and panNEN was not completely clear in the univariable model. Compared to never smokers, no significant association was found among former smokers (OR = 0.99, 95% CI: 0.84–1.18) or current smokers (OR = 1.40, 95% CI: 0.99–1.98). Pack-years of smoking was also not associated with the odds of panNEN. Compared to never smokers, the ORs (95% CIs) for <10 pack-years, 10–19 pack-years and ≥20 pack-years of smoking were 0.99 (0.78–1.26), 0.94 (0.67–1.29) and 0.94 (0.74–1.19), respectively. In addition, no association was found between pack-years of smoking within smoking categories among former smokers. However, an association was found among current smokers with <10 pack-years of smoking (OR = 4.32, 95% CI: 1.40–16.00), but not current smokers with 10–19 pack-years of smoking (OR = 1.15, 95% CI: 0.39–3.12) or current smokers with ≥20 pack-years of smoking (OR = 1.13, 95% CI: 0.66–1.90).

A personal history of T2DM was associated with 77% higher odds of panNEN in the univariable model (OR = 1.77, 95% CI: 1.45–2.16) (Table 1). In terms of duration of T2DM, individuals with new-onset T2DM had 3-fold higher odds of panNEN (OR = 2.96, 95% CI: 2.19–4.03), but no association was found for those with 1–4 years’ duration of T2DM (OR = 1.23, 95% CI: 0.74–1.98) or longstanding T2DM (OR = 1.27, 95% CI: 0.95–1.69), compared to those without T2DM. We also found an inverse association between taking 1 and 2 aspirin pills a day (OR = 0.79, 95% CI: 0.66–0.95) but not ≥3 aspirin pills daily (OR = 0.78, 95% CI: 0.54–1.11) and odds of panNEN, as compared to non-regular aspirin users. Furthermore, an inverse association was found between alcohol use and panNEN odds in the univariable model (OR = 0.50, 95% CI: 0.41–0.63, ever *vs* never). A similar inverse association was found for the number of alcoholic drinks consumed per day. Compared to non-alcohol users, ORs (95% CIs) for those who consume <1 alcoholic drink/day, 1–2 drinks/day and ≥3 drinks/day were 0.50 (0.40–0.64), 0.62 (0.46–0.84) and 0.35 (0.24–0.50), respectively.

Table 2 presents results for both the minimally adjusted and fully adjusted models. We summarize here results of the fully adjusted models, as they account for the effects of multiple potential confounders. The results do not show an association between race and the odds of panNEN (OR = 0.93, 95% CI: 0.56–1.52, other *vs* White). Obesity was also not associated with odds of panNEN (OR = 1.05, 95% CI: 0.83–1.32, BMI ≥30 kg/m^2^ *vs* <24.9 kg/m^2^; OR_5-unit increase_ = 0.99, 95% CI: 0.92–1.07; OR_continuous_ = 1.00, 95% CI: 0.98–1.01). A first-degree family history of pancreatic cancer was again non-significantly associated with elevated odds of panNEN (OR = 1.44, 95% CI: 0.96–2.14). Former smoking (OR = 1.18, 95% CI: 0.98–1.42) and current smoking (OR = 1.30, 95% CI: 0.89–1.87) were both not associated with the odds of panNEN, compared to never smoking. Pack-years of smoking was also not associated with panNEN, nor was pack-years of smoking within smoking categories (Table 2). Personal history of T2DM was associated with 71% higher odds of panNEN in the fully adjusted model (OR = 1.71, 95% CI: 1.37–2.14). New-onset T2DM was also associated with a nearly 3-fold higher odds of panNEN (OR = 2.65, 95% CI: 1.92–3.69), but not 1–4 years’ duration of T2DM (OR = 1.28, 0.76–2.12) or longstanding T2DM (OR = 1.29, 95% CI: 0.94–1.75). In the fully adjusted model, aspirin use was not associated with panNEN. The ORs (95% CIs) for taking 1–2 aspirin pills daily and ≥3 aspirin pills daily were 1.04 (0.85–1.27) and 1.06 (0.73–1.52), respectively. Ever use of alcohol was again inversely associated with the odds of panNEN in the fully adjusted model (OR = 0.52, 95% CI: 0.42–0.66, ever *vs* never). We further observed an inverse association between panNEN and the number of alcoholic drinks consumed per day, with ORs of 0.52 (95% CI: 0.42–0.69) for consuming <1 drink/day, OR of 0.65 (95% CI: 0.47–0.88) for 1–2 drinks/day and OR of 0.36 (95% CI: 0.25–0.53) for ≥3 drinks/day. These results are similar to those observed in the sensitivity analyses, where we excluded participants with missing data (Supplementary Table S1).

## Discussion

This is the largest study to date on risk factor assessment for panNEN. We investigated whether known risk factors of PDAC are also associated with the odds of being diagnosed with nonfunctional sporadic panNEN. Our results show that overall T2DM and new-onset T2DM are associated with higher odds of panNEN. A positive first-degree family history of pancreatic cancer was non-significantly associated with higher odds of panNEN. Alcohol intake, even at three or more drinks per day, was inversely related to panNEN. This stands in contrast to the known association between alcohol intake and PDAC (Genkinger *et al.* 2009, Wang *et al.* 2016, Antwi *et al.* 2017). In addition, unlike PDAC, we did not find any association between BMI, cigarette smoking or aspirin use and panNEN risk. These results help clarify the risk factors associated with panNEN development and are important for targeted prevention, risk stratification and distinguishing between panNEN and PDAC risk factor profiles.

Our finding of an association between overall T2DM and panNEN is consistent with results from previous studies (Hassan *et al.* 2008, Halfdanarson *et al.* 2014, Ben *et al.* 2016, Valente *et al.* 2017, Giraldi *et al.* 2021, Feola *et al.* 2022, Hernandez-Rienda *et al.* 2022, Bogaards *et al.* 2023). Here, we add evidence of separate assessments of new-onset T2DM, T2DM duration of 1–4 years and longstanding T2DM. For these, we only found a significant association for new-onset T2DM, suggesting a moderation of risk with longer duration of T2DM, a phenomenon also observed in PDAC (Bosetti *et al.* 2014, Antwi *et al.* 2017). However, because a significant association was observed only with new-onset T2DM when evaluating T2DM duration, it is entirely plausible that T2DM could be a consequence, as opposed to a risk factor, of panNEN. Essentially, the presence of panNEN may induce the occurrence of T2DM, and this needs to be verified in larger studies, perhaps through a consortium effort with pooled data across multiple institutions. The results further showed a non-significant elevated odds of panNEN in participants with a first-degree family history of pancreatic cancer, which is in line with results from four other studies reporting a significant association between family history of pancreatic cancer or overall cancer and panNEN risk (Hassan *et al.* 2008, Capurso *et al.* 2009, Ben *et al.* 2016, Giraldi *et al.* 2021). Our non-significant elevated risk for family history of pancreatic cancer may be due to the focus on sporadic panNEN, which led to the exclusion of individuals with clinical diagnosis of known genetic syndromes that predispose to panNEN. Furthermore, we found that even at three or more drinks a day, alcohol intake is inversely associated with panNEN after adjusting for multiple potential confounders. This is consistent with our previous case-control study that showed a 44% panNEN risk reduction among alcohol users (OR = 0.56, 95% CI: 0.38–0.82, ever *vs* never) (Halfdanarson *et al.* 2014). However, two smaller case-control studies have reported a higher risk of panNEN among alcohol users: one from China (OR = 1.87, 95% CI: 1.01–3.51, ≥30 g/day *vs* non-users) (Ben *et al.* 2016) and the other from Italy (OR = 4.8, 95% CI: 2.4–9.5, >21 drinks/week *vs* non-users) (Capurso *et al.* 2009). Of note, alcohol use has been associated with lower risk of other malignancies, including renal cancer (Parker *et al.* 2002, Song *et al.* 2012, Antwi *et al.* 2018*b*) and non-Hodgkin lymphoma (Tramacere *et al.* 2012, Psaltopoulou *et al.* 2018), but the potential mechanism(s) are unknown. Thus, additional prospective studies would help clarify the association between alcohol intake and panNEN risk.

Obesity is an established risk factor for PDAC (Bracci 2012, Klein 2021), but prior studies on the association between obesity and panNEN have reported conflicting findings (Capurso *et al.* 2009, Halfdanarson *et al.* 2014, Valente *et al.* 2017, Giraldi *et al.* 2021, Feola *et al.* 2022). In a clinic-based case-control study that included 309 panNEN cases and 602 controls, being obese was associated with higher odds of panNEN in univariable analysis (OR = 1.65, 95% CI: 1.11–2.45) (Halfdanarson *et al.* 2014). Another case-control study of 75 panNEN cases and 210 controls recruited from three centers in Italy reported a higher odds of panNEN among participants with obesity (OR = 1.98, 95% CI: 1.11–3.52) after adjusting for only age and sex (Feola *et al.* 2022). A separate multicenter study of 100 panNEN cases and 248 controls did not find an association between obesity and panNEN (Giraldi *et al.* 2021), and neither did two other studies (Capurso *et al.* 2009, Valente *et al.* 2017). In the present study, we found that higher BMI was not associated with panNEN risk after controlling for multiple potential confounders, including family history of pancreatic cancer, alcohol use and T2DM, which were not adjusted for in any of the prior studies cited above.

We (Antwi *et al.* 2016, 2018*a*, 2022) and others (Lynch *et al.* 2009, Wolpin *et al.* 2009, Bosetti *et al.* 2012) have shown that cigarette smoking is associated with higher risk of PDAC. However, in the present study, we did not find any association between cigarette smoking and panNEN risk. Of six prior studies that investigated the association between smoking and panNEN risk, five studies also did not find an association (Hassan *et al.* 2008, Capurso *et al.* 2009, Halfdanarson *et al.* 2014, Valente *et al.* 2017, Giraldi *et al.* 2021). Only one small study from China reported an association between heavy smoking (≥21 pack-years) and panNEN risk (Ben *et al.* 2016). Besides having a larger sample size in the present study, we assessed associations for smoking category (never, former or current), pack-years of smoking and pack-years of smoking within smoking category and found no association between any of these variables and risk of panNEN after adjusting for potential confounders. Thus, the overall evidence does not support an association between smoking history and panNEN. In addition, to our knowledge, this is the first study to investigate the association between aspirin use and panNEN risk, and we did not find an association for aspirin use after fully adjusting for potential confounders.

Our study population is comparable to most published studies. The mean age at panNEN diagnosis in our study was 59 years, the majority (59%) of cases were men and predominantly White (97%), all of which are consistent with most prior studies (Hassan *et al.* 2008, Capurso *et al.* 2009, Valente *et al.* 2017, Feola *et al.* 2022). However, one study performed among the Han Chinese population showed a much lower mean age of panNEN diagnosis of 50 years, with a higher proportion of women (55%) having panNEN than men (Ben *et al.* 2016). Another study from Italy also showed a higher proportion of women (54%) with panNEN than men (Giraldi *et al.* 2021), which differs from our study population. Furthermore, our study participants were recruited over a 24-year period (2000–2023). It is worth noting that pathological classifications of panNENs have changed multiple times over the past 24 years, with panNENs currently classified as well-differentiated grade 1 (Ki-67 <3%), well-differentiated grade 2 (Ki-67 3–20%), well-differentiated grade 3 (Ki-67 >20%) or neuroendocrine carcinomas (poorly differentiated tumor with Ki-67 >20%), as reviewed in detail elsewhere (Rindi *et al.* 2022, Helderman *et al.* 2024). The Ki-67 was not consistently used in practice at Mayo Clinic before 2018.

Our study has several strengths and limitations. Major strengths include being the largest study to date on panNEN, a rare malignancy. Our single-institution series of over 900 nonfunctional sporadic panNEN cases accrued over two decades using a single protocol and standardized risk factor questionnaire minimizes heterogeneity in case ascertainment and risk factor assessment. Each panNEN diagnosis was confirmed by a careful review of pathology data, and electronic medical records were available on all patients. This allowed for the exclusion of functional panNEN cases to minimize sample heterogeneity. We also excluded patients identified clinically as carriers of pathogenic germline variants in *MEN1* or *VHL*, who frequently develop panNEN and could introduce some bias in the study. However, it is important to note that there may be patients who harbor germline pathogenic or likely pathogenic variants in other genes (e.g., BRCA1/2) that could potentially predispose to panNEN, but these were not tested clinically in the present study. Furthermore, we performed detailed assessments of cigarette smoking history, T2DM, and aspirin use in relation to panNEN risk and adjusted for the effects of multiple potential confounders. Limitations include the predominantly White population, which limits generalizability to other racial groups. The retrospective case-control design, with its inherent potential for recall and selection biases, is also a limitation. Our pancreas cancer registry addresses selection bias by utilizing an ultra-rapid case recruitment process that ensured that 55% of cases were enrolled within 30 days of diagnosis, and we had a high response rate (69%) among our cases. We had missing data on some variables, and thus, we performed a sensitivity analysis excluding individuals with missing information, and the results were consistent with the overall analysis. While we cannot rule out the possibility of differential recall of risk factors between the cases and controls, it is important to note that the cases and controls were recruited from the same health system with similar referral patterns. Although controls were patients without a history of cancer and were recruited from primary care clinics, they were unaware of the outcome of interest of the study. Therefore, any potential differences in recall are likely to be non-differentially related to panNEN status, which tends to attenuate effect estimates toward the null value (Rothman *et al.* 2008). Finally, we cannot rule out the effects of residual confounding or confounding by unmeasured factors.

In summary, our study shows that overall T2DM and new-onset T2DM are associated with a higher risk of nonfunctional sporadic panNEN. These associations are shared between PDAC and panNEN. However, unlike PDAC, alcohol intake was inversely related to panNEN. In addition, we did not find an association between BMI, cigarette smoking or aspirin use with panNEN risk. These findings shed light on the similarities and differences in risk factor profiles between panNEN and PDAC and could inform targeted strategies for risk prevention. Additional prospective studies with greater racial diversity would help further characterize the risk factors of panNEN.

## Supplementary materials



## Declaration of interest

The authors declare that there is no conflict of interest that could be perceived as prejudicing the impartiality of the work reported.

## Funding

The study was supported in part by NIHhttps://doi.org/10.13039/100000002 grant P50 CA102701 (Mayo Clinic SPORE in Pancreatic Cancer) and the Lustgarten Foundationhttps://doi.org/10.13039/100005979 for Pancreatic Cancer Research. Funders had no role in study design, data collection, analysis or manuscript writing.

## Author contribution statement

SC wrote the first draft of the manuscript and SOA provided critical review and oversight of the study. All authors contributed to the planning, analyses or manuscript writing and they all reviewed and approved the final version of the manuscript.

## Data availability

The data can be made available to researchers upon request to Dr Samuel O Antwi (Antwi.samuel@mayo.edu). Institutional policies, including ethical and legal restrictions, apply to these data.

## Ethical approval and participant consent

The study was approved by the Mayo Clinic Institutional Review Board (IRB #: IRB 21–005418). All participants provided informed consent.
