# Supplementary material for: Discordant risk factors between pancreatic neuroendocrine neoplasms and pancreatic ductal adenocarcinoma
Source: Endocr Relat Cancer. 2025 Mar 1;32(4):e240142. doi: 10.1530/ERC-24-0142 (PMC11896652; doi:10.1530/ERC-24-0142)
Supplement: Supplementary file 1 [file supplementary_materials.pdf]

Table S1. Sensitivity Analysis: Excluding data with unknown alcohol use history.

Risk factors	panNEN Cases (n=674) *N* (%)	Cancer-free Controls (n=1666) *N* (%)	Fully-adjusted OR (95% CI)[a]
Race			
White	659 (98)	1626 (98)	(ref)
Other	15 (2)	40 (2)	0.84 (0.44-1.53)
BMI, kg/m^2			
≤24.9	168 (25)	508 (31)	(ref)
25-29.9	272 (40)	694 (42)	1.12 (0.89-1.42)
≥30	234 (35)	464 (28)	1.18 (0.92-1.53)
Continuous (mean ± SD)	28.9 ± 5.6	28.0 ± 5.7	1.01 (0.99-1.02)
Family history of pancreatic cancer[b]			
No	635 (94)	1600 (96)	(ref)
Yes	39 (6)	66 (4)	1.46 (0.96-2.21)
Smoking history			
Never	400 (59)	990 (59)	(ref)
Former	238 (35)	601 (36)	1.01 (0.83-1.23)
Current	36 (5)	75 (5)	1.16 (0.75-1.75)
Pack-years of smoking			
Never smokers	435 (65)	1069 (64)	(ref)
< 10	95 (14)	231 (14)	1.11 (0.84-1.45)
10-19	46 (7)	121 (7)	1.00 (0.69-1.43)
≥20	98 (14)	245 (15)	0.98 (0.74-1.28)
Pack-years of smoking within smoking category			
Never	435 (65)	1069 (64)	(ref)
Former			
< 10	91 (14)	227 (14)	1.09 (0.83-1.43)
10-19	43 (6)	111 (7)	1.03 (0.70-1.49)
≥20	81 (12)	206 (12)	0.95 (0.71-1.28)
Current			
< 10	4 (0.6)	4 (0.2)	2.05 (0.47-8.98)
10-19	3 (0.4)	10 (0.6)	0.70 (0.15-2.38)
≥20	17 (3)	39 (2)	1.10 (0.60-1.95)
History of T2DM			
No	506 (75)	1435 (86)	(ref)
Yes	168 (25)	231 (14)	1.95 (1.53-2.47)
Duration of T2DM[c]			
No T2DM	506(75)	1435 (86)	(ref)
≤ 1 year	83 (12)	59 (4)	3.71 (2.58-5.36)
1-4 years	22 (3)	42 (3)	1.40 (0.81-2.38)
5+ years	63 (9)	130 (8)	1.31 (0.94-1.82)

Table S1. (Continued)

Risk factors	panNEN Cases (n=674) N (%)	Cancer-free Controls (n=1666) N (%)	Fully Adjusted OR (95% CI)[a]
Number of aspirin pills taken regularly			
Non-users/< 1 per month	443 (66)	1055 (63)	(ref)
1-2 per day	190 (28)	502 (30)	0.97 (0.78-1.19)
≥ 3 per day	41 (6)	109 (7)	1.01 (0.68-1.47)
Ever alcohol use			
No	173 (26)	247 (15)	(ref)
Yes	501 (74)	1419 (85)	0.53 (0.42-0.66)
Alcoholic drinks per day			
Non-users	173 (26)	247 (15)	(ref)
< 1	349 (52)	984 (59)	0.52 (0.41-0.65)
1-2	103(15)	236 (14)	0.68 (0.50-0.93)
≥3	49 (7)	199 (12)	0.39 (0.26-0.56)

[a]Adjusting for matching factors: Age (continuous), sex, regions of residence (Midwest, other), race (white, other), BMI (continuous), family history of pancreas cancer (yes, no), smoking status (never, former, current), type II diabetes mellitus (yes, no), and alcohol use (yes, no). We did not adjust for any of the BMI variables in models examining associations for BMI. Similarly, we did not adjust for any of the diabetes variables in models examining associations for diabetes; same with alcohol, smoking, and aspirin use.

[b]Family history of pancreatic cancer (either pancreatic ductal adenocarcinoma or pancreatic neuroendocrine neoplasm)

[c]Duration of T2DM prior to the diagnosis of pancreatic neuroendocri ne neoplasm for cases or prior to recruitment for controls.

Abbreviations: BMI, body mass index; CI, confidence interval; T2DM, type II diabetes mellitus; OR, odds ratio; panNEN, pancreatic neuroendocrine neoplasm
